# In Vitro Biocompatibility Assessment of a Novel Membrane Containing Magnesium-Chitosan/Carboxymethyl Cellulose and Alginate Intended for Bone Tissue Regeneration

**DOI:** 10.7759/cureus.54597

**Published:** 2024-02-21

**Authors:** Deepika Subramaniam, Saravanan Sekaran

**Affiliations:** 1 General Pathology, Saveetha Dental College and Hospitals, Saveetha Institute of Medical and Technical Sciences, Saveetha University, Chennai, IND; 2 Prosthodontics, Saveetha Dental College and Hospitals, Saveetha Institute of Medical and Technical Sciences, Saveetha University, Chennai, IND

**Keywords:** osteoblasts, biocompatibility, alginate, chitosan, health care, bone tissue engineering

## Abstract

Bone tissue engineering (BTE) is an emerging interdisciplinary field that aims to develop new strategies and materials for repairing, regenerating, or replacing damaged bone tissues. This field combines engineering, biology, and medicine principles to create functional bone tissues in the laboratory and in vivo. The main goal of BTE is to create biological substitutes that mimic the structure, function, and properties of natural bone tissue, thereby promoting the regeneration of bone defects caused by trauma, disease, or aging. In this study, we developed a biocomposite membrane using magnesium-chitosan, carboxymethyl cellulose, and alginate through a simple cast drying method. The biocompatibility of the membrane was evaluated using human osteoblastic cells, and it was found to be nontoxic to these cells. Both metabolic activity measurements (24 and 48 hours) and the lactate dehydrogenase release assay (72 hours) indicated that the membrane was biocompatible and did not exert significant toxic effects. These results suggest that the developed biocomposite membrane has the potential to be used as a safe and effective biomaterial for various biomedical applications, such as BTE, wound healing, and drug delivery. Further studies are warranted to explore the full potential of this membrane and its performance in different biological environments.

## Introduction

Bone tissue engineering (BTE) is an emerging field that aims to develop strategies for regenerating damaged or lost bone tissue. The ultimate goal is to create functional bone tissue capable of replacing damaged or lost bone due to trauma, disease, or congenital abnormalities, addressing the limitations of current treatment options [1]. This innovative field combines biological and engineering principles, utilizing biomaterials, stem cells, and growth factors to create scaffolds supporting the growth of new bone tissue [2]. The scaffold acts as a template, with stem cells and growth factors providing signals for bone formation [3]. Upon the formation of new bone tissue, the scaffold may degrade or remain in place for structural support. A significant challenge in BTE is developing suitable biomaterials that are biocompatible, biodegradable, and possess appropriate mechanical properties. Various biomaterials, including ceramics, polymers, and composites, have been investigated [4], with recent exploration of new material combinations.

Chitosan (CS), a naturally occurring biopolymer derived from chitin, has garnered attention in BTE due to its biocompatibility, swelling ability, biodegradability, and support for cell adhesion and proliferation [5-7]. CS’ amino, carboxyl, and hydroxyl groups play crucial roles in its biological activities [8,9], and its structure is similar to glycosaminoglycans. Magnesium (Mg), abundantly present in the human body, has been utilized in biomaterials for orthopedic implants due to its advantageous mechanical properties and ability to promote bone growth. Mg is involved in osteoblast differentiation, cellular adhesion, inhibition of osteoclast activity, and immunomodulation [10-14].

Carboxymethyl cellulose (CMC), a water-soluble, biocompatible, and biodegradable biopolymer derived from cellulose, has potential in BTE. CMC can be used as a scaffold material to support bone tissue growth and regeneration, promoting cell adhesion and proliferation. It can also be combined with other biomaterials, such as hydroxyapatite (HA), to enhance scaffold osteoconductivity [15-19]. CMC has been explored as a carrier for delivering bioactive cues, such as growth factors and drugs, to bone repair sites, allowing controlled release to enhance the healing process. Strategies like cross-linking and blending with other polymers have been employed to improve the mechanical properties of CMC-based scaffolds.

Alginate, a biopolymer derived from brown seaweed, is biocompatible, low-toxic, and easily gelates in the presence of divalent cations like calcium [20]. Alginate-based scaffolds, fabricated using techniques such as three-dimensional (3D) printing, electrospinning, and freeze drying, support bone cell growth and differentiation, providing mechanical support to newly formed bone tissue [21].

In our study, the objective was to fabricate a biocomposite membrane comprised of CS chelated with Mg, CMC, and alginate. We assessed its biocompatibility through in vitro experiments, conducting cytotoxicity tests using indirect 3-(4,5-dimethylthiazol-2-yl)-2,5-diphenyltetrazolium bromide (MTT) assays and lactate dehydrogenase (LDH) measurements.

## Materials and methods

This study was conducted at Saveetha Dental College and Hospitals, Chennai, India.

Materials 

CS, alginate, CMC, and Mg chloride were purchased from Sisco Research Laboratories Pvt. Ltd (SRL; Mumbai, India). Cell culture media (Dulbecco’s Modified Eagle Medium, DMEM), MTT reagent, fetal bovine serum, and the LDH assay kit were purchased from the National Centre for Cell Science (Pune, India).

CS-Mg chelated powder preparation

CS (2% w/v) was dissolved in 1% (v/v) acetic acid for two hours under constant stirring to obtain a clear solution. A total of 0.25 M Mg chloride solution was added to the above mixture and refluxed at 60 °C for four hours following the addition of 1 M NaOH solution to adjust the pH to 7.4. After the reflux, the precipitate was sterile filtered, washed with deionized water three times, and lyophilized to obtain the CS-Mg powder.

Biocomposite membrane preparation

CS-Mg 1% (w/v) was prepared by dissolving 100 mg of CS-Mg powder in 1 M acetic acid for one hour under stirring conditions. Then 1% CMC and 1% alginate (w/v) were added to the above mixture and stirred overnight. The solution was then poured into the Petri dish and dried at room temperature. The membrane was then neutralized with a 1 M NaOH solution, washed several times with deionized water, and dried at room temperature. The membrane was then peeled off and stored under a desiccator until further use.

Biocompatibility assessment using the MTT assay and the LDH assay

The MTT assay and LDH assay were performed to assess biocompatibility, as reported in one of our earlier studies [9]. The biocomposite membrane was sterilized by soaking it in 70% ethanol for 30 minutes and then rinsing it three times with sterile phosphate-buffered saline. The membranes were immersed in DMEM for 24 hours at 37 °C. Next, the solution was filtered (known as conditioned medium) and treated with human MG-63 cells for 24 and 48 hours in each well of a 96-well cell culture plate (10,000 cells in each well). The conditioned medium was treated with the cells at different concentrations (25%, 50%, 75%, and 100%). The indirect MTT assay was based on the ISO 10993 standard protocol for biological evaluation of medical devices. At the end of the treatment period, the cell culture medium was removed (stored for LDH assay), and 100 μL of MTT reagent was added to each well. The plate was then incubated for four hours at 37 °C in a humidified cell culture incubator with 5% CO_2_. MTT reagent from each well was removed, and 100 μL of DMSO was added to each well. After one hour of incubation, the absorbance was measured at 570 nm using a microplate reader, and a graph was plotted. The supernatant (medium collected following treatment) was collected. LDH release was measured according to the manufacturer’s instructions (Abcam LDH kit, Abcam Limited, Cambridge, United Kingdom), and a graph was plotted.

Statistical analysis

The tests were carried out three times, and the outcomes were expressed as the average value accompanied by the SD. To assess statistical significance, a Student’s t-test was executed using the IBM SPSS Statistics for Windows, Version 29.0.1 (Released 2023; IBM Corp., Armonk, NY, USA). A significance level below 0.05 was considered to be statistically significant.

## Results

Membrane preparation

**Figure 1:**
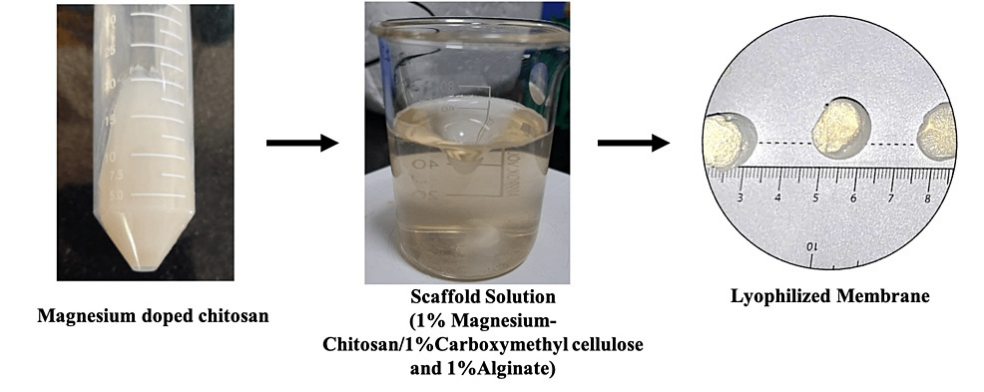
Pictorial representation indicates the appearance of scaffolding solution and lyophilized membrane

**Figure 2:**
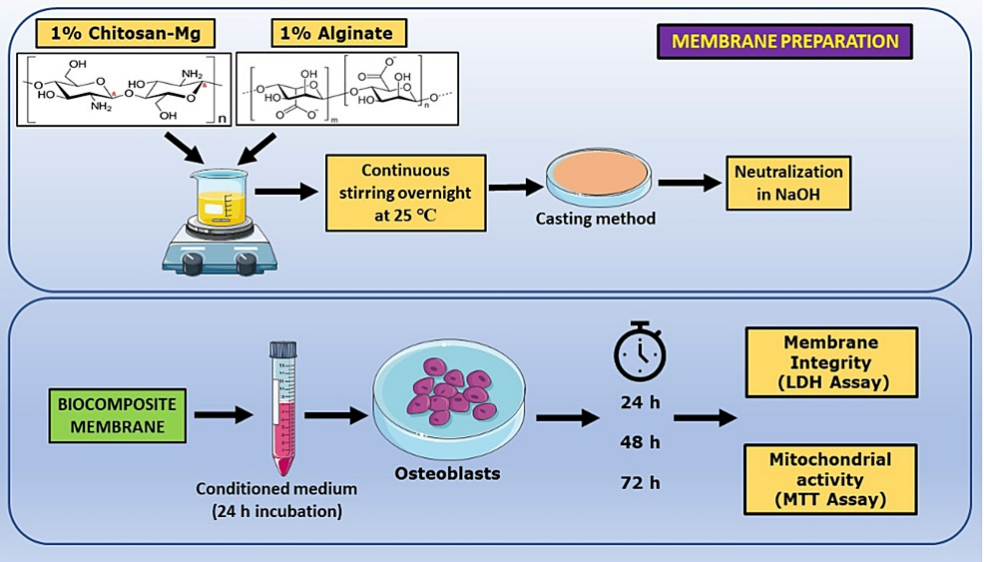
Overall schematic representation of the methodology involved in the preparation of biocomposite membrane and its biocompatibility assessment The top panel represents the steps involved in membrane preparation, and the bottom panel portrays the methods used to assess the biocompatibility of the membrane. CMC was added along with alginate powder to the CS-Mg solution to form the biocomposite membrane. CMC, carboxymethyl cellulose; CS, chitosan; LDH, lactate dehydrogenase; Mg, magnesium; MTT, 3-(4,5-dimethylthiazol-2-yl)-2,5-diphenyltetrazolium bromide Image credit: Saravanan Sekaran

The biocompatibility of membranes for BTE is a critical aspect of the development of effective bone graft substitutes. The evaluation of biocompatibility through appropriate in vitro and in vivo assays is essential for the successful translation of these materials into clinical applications. In the current study, we have investigated the biocompatibility of the CS-Mg/CMC/alginate membrane by assessing (a) metabolic activity via the MTT assay and (b) membrane integrity via the LDH leakage assay. The overall methodology is represented in Figure 1 and Figure 2. The scaffolding solution was lyophilized to obtain porous scaffolds and neutralized to pH 7.0 for further in vitro studies.

MTT assay

**Figure 3:**
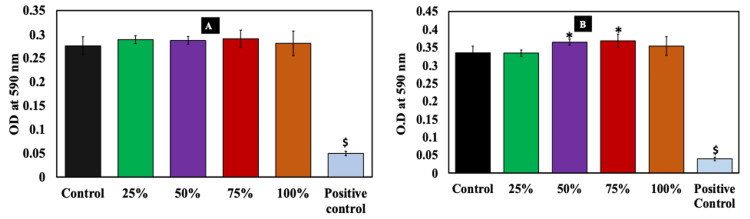
Metabolic activity assessment using indirect MTT assays (A) and (B) for 24 and 48 hours Cells were treated with different percentages of conditioned medium (25-100%) plotted on the x-axis. Control wells have only cells without any biocomposite membrane, and cells treated with 0.1% Triton X-100 were denoted as positive controls for the MTT assay. Results indicated that the biocomposite membrane was found to be potentially biocompatible with no discrete toxicity. * indicates a significant increase compared to control, while $ indicates a significant decrease compared to control (p ≤ 0.05; n = 3). MTT, 3-(4,5-dimethylthiazol-2-yl)-2,5-diphenyltetrazolium bromide; OD, optical density

Biomaterial-based constructs intended for in vivo applications should be cytofriendly and should not exert any toxicity on mammalian cells. Therefore, prior testing of its biocompatibility is essential for promoting its clinical applications. To assess the biocompatibility, human MG-63 cells were treated with the conditioned medium obtained from the membrane, and an MTT assay was carried out. Figure 3A-3B depicts the optical density (OD) measurements following the MTT assay for 24 and four hours, respectively. The results indicated that at all the tested concentrations, there was no significant change in the OD when compared to the control. This clearly indicated the biocompatibility of the membrane.

LDH measurements

**Figure 4:**
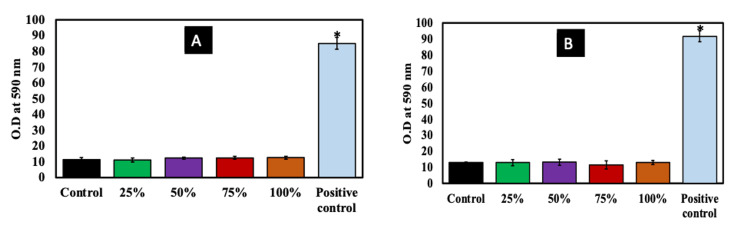
Membrane integrity assessment using LDH assays (A) and (B) for 48 and 72 hours Cells were treated with different percentages of conditioned medium (25-100%) as represented in the x-axis. The OD is plotted on the y-axis. Control wells have only cells without any biocomposite membrane, and cells treated with 100 μM H_2_O_2_ served as positive controls. The results show that there was no significant change in membrane integrity, as seen by the minimal LDH leak from all the groups. The experiment was performed in triplicate (n = 3). * indicates a significant increase compared to the control. LDH, lactate dehydrogenase; OD, optical density

We observed that the MTT assay concluded that there was no significant toxicity to the human osteoblastic cells. The cell membrane is the preliminary site of interaction for biomaterials and nanomaterials. Thus, we were interested in identifying whether there was any change in the cell membrane integrity following exposure to the conditioned medium obtained from the biocomposite material. Figure 4A-4B indicates the biocomposite membrane in the conditioned medium assessed using the LDH assay for 24 and 72 hours, respectively. The results indicate that the LDH leak from the cell membrane is minimal at all concentrations (25-100%) of the biocomposite membrane compared to the control.

## Discussion

Large bone defects, referred to as critical-sized defects, present a challenge as they do not naturally heal within the body, and traditional bone grafts have limitations. To address this issue, BTE has emerged as a promising approach, utilizing 3D bioprinted scaffolds. These scaffolds incorporate living cells and growth factors, aiming to mimic the natural bone’s structure and properties. Researchers have explored various biomaterials, whether natural or synthetic polymers, along with different cell types and growth factors for scaffold bioprinting. Nevertheless, a key obstacle that remains is the development of scaffolds that not only meet the structural and mechanical criteria of native bone but also promote vascularization, a crucial factor for successful bone regeneration. The scaffolds should not exert any significant toxicity on mammalian cells for their effective clinical translation. Therefore, we decided to evaluate biocompatibility by assessing the metabolic activity and membrane integrity of MG-63 cells grown on the synthesized Mg-CS/CMC/alginate membrane. The results clearly indicated that at all tested concentrations, the membrane did not alter the metabolic activity of osteoblasts at both 24 and 48 hours, and there were no alterations in the cell membrane integrity at the end of 48 and 72 hours.

CS stands out as the most extensively studied natural polymer compared to other available options like alginate, collagen, hyaluronic acid, collagen, silk, and fibrin [3]. This preference is due to several advantageous properties of CS, including its negligible foreign body response, ability to biodegrade into nontoxic by-products, intrinsic antibacterial activity, and cost-effectiveness. In our study, we formed a complex coacervate by blending CS with a negatively charged polymer, CMC, and alginate. Despite having similar structures, CS, alginate, and CMC have opposite electric charges, which facilitate a robust interaction between them, leading to the formation of a polyelectrolyte network structure [22]. In the literature, the combination of CS and CMC is widely explored and found to be biocompatible. For instance, human osteoblastic cells responded positively to the CS/CMC/m-WS scaffolds, indicating their cytofriendly nature. The scaffolds demonstrated osteogenic potential, as evidenced by calcium deposition and the expression of a specific microRNA, pre-mir-15b, associated with osteoblast function [23]. In another research study, a composite scaffold made of sodium alginate, CS, and HA was created and evaluated. The study confirmed that the scaffold was biocompatible and did not exhibit any toxicity toward bone marrow-derived mesenchymal stem cells [24].

We have also chelated Mg into CS with a view to improving its osteogenic properties. Mg plays a vital and diverse role in numerous molecular and cellular functions, impacting energy metabolism, regulating cellular signal transduction, contributing to tooth and bone mineralization, and influencing cell migration, differentiation, and proliferation [25]. The inclusion of Mg ions is likely to alter the biocompatibility of the biocomposite membrane. In various other investigations, Mg ions have been shown to enhance cell viability, encourage cellular differentiation and migration, and induce gene expression [26]. CS-Mg-based biocomposite scaffolds were found to be biocompatible with 3T3 fibroblasts. In our study, the membrane containing Mg was found to be biocompatible [27]. Based on the current ISO standards (Part 5), if the cellular viability is greater than 75%, it can be denoted as safe with no toxicity for medical devices. In our study, the membrane resulted in more than 75% cell viability, indicating its safety.

Limitations

The current study is limited only to the biocompatibility of the prepared biocomposite membrane under in vitro conditions. However, a long-term study on the biocompatibility and molecular mechanisms behind the osteogenic role of the scaffold is essential to further warrant its in vivo applications. One major limitation of the current study is the control over the release of Mg ions from the CS-Mg composite. In vitro studies may not exactly recapitulate the microenvironment observed in vivo, including blood flow, angiogenesis, and 3D microarchitecture. Therefore, further investigations are required to exactly assess the biocompatibility using a suitable animal model. Application of this scaffold in an in vivo model needs careful consideration of the site of implantation and the desired time point that needs to be assessed to study bone formation.

## Conclusions

Overall, the results of this study suggest that the Mg-CS/CMC/alginate membrane is biocompatible and can promote cell adhesion and osteogenic differentiation. The membrane was found to exert no significant toxicity toward osteoblasts, with no discrete signs of membrane damage or metabolic functions. These findings support the use of the membrane as a potential material for biomedical applications, specifically in BTE. However, it should be noted that this study only evaluated the in vitro biocompatibility of the Mg-CS/CMC/alginate membrane. Further in vivo studies are necessary to assess its biocompatibility, degradation rate, and potential use in tissue engineering applications. Additionally, the long-term effects of the membrane on surrounding tissues need to be evaluated to ensure its safety and efficacy.
